# Semi-Annual Meeting of the Esculapian Society

**Published:** 1854-06

**Authors:** Charles Johnston, S. W. Thompson

**Affiliations:** President; Marshall, Illinois; Secretary; Marshall, Illinois


*Semi-Annual Meeting of the Æsculapian Society.*

MARSHALL. Illinois, May 31st, 18[illegible]

THE semi-annual meeting of the Æsculapian Society t[illegible] to-day. The assembly being called to order, it was fo[illegible] Secretary was absent, when Dr. S. W. THOMPSON [illegible] cretary *pro tem.* Two of the Censors not be[illegible]

D. MITCHELL and S. W. THOMPSON were selected to fill the vacancy.

The following named gentlemen were then reported as applicants for membership:

Drs. York, Davis, McClure, Tenbrook, Churchill, Pool, Hartman, McCord, Holmes and Maxwell; and after receiving the sanction of the Censors, were unanimously elected.

The Committee on the revision of the constitution not being able to report, the following were designated to fill their places:

Drs. York, Tenbrook, McClure, and Davis.

The reading of Essays and Reports being next in order, Dr. H. F. R. Payne read as an essay on the Use of Opium. Dr. S. W. Thompson presented an essay on the nature of Milk Sickness, which gave rise to some discussion. Dr. Duncan read an essay on Dropsy; after which, the President, Dr. Johnson, addres[illegible] the meeting on the subject of Empiricism.

The Society then adjourned to meet at 7½ o'clock, P. M., in the Congregational Church.

In the evening a large audience assembled in the church, where they listened attentively to an address by Dr. Payne. The speaker had chosen for his subject, the "[illegible]ure and Character of the True Physician," which he ably discussed.

---

### MORNING SESSION.

JUNE 1st. The minutes of the previous day were read and adopted.

Reports of cases being called for, Dr. J. D. Mitchell, read a case of Disease of the Heart, with Autopsy. Dr. F. R. Payne reported a case of Hepatic Abscess. Dr. S. W. Thompson submitted a report of a case of Opthalmia, with suppuration in the globe of the eye.

On motion it was ordered that the essays and reports of cases, be placed in the hands of the secretary, and forwarded for publication.

The case of Dr. I. A. Powell, who was charged with violating articles XIII and XIV of Medical Ethics, was then taken into consideration; and on motion of Dr Thompson, the said Powell was unanimously declared *expelled* from this Society.

On motion, *Resolved*, That a Committee of six members be appointed, to report at the next meeting, the most feasible plan for the suppression of Quackery; and whether it is expedient to legislate on the manufacture and vending of nostrums and quack medicines.

Drs. F. R. Payne, Churchill, Pool, Johnson, Mitchell and Davis, were elected such committee.

A committee of seven was then appointed to canvass the subject of Medical Science, and to endeavor to elevate the standard of the medical profession in their several localities.

The following gentlemen constitute this committee: Drs. F. R. Payne, Johnson, York, Logan, Maxwell, Thompson and Mitchell.

On motion, a committee of three were appointed, to solicit a copy of Dr. F. R. Payne's address for publication; and, if published the committee was instructed to furnish each member of the society with a copy of the same.

On motion, *Resolved*, That the proceedings of this Society be published in the Marshal, Paris, Charleston, Vincennes, and Mount Carmel papers and North-Western Medical and Surgical Journal.

Drs. Davis, McClure, Mitchell and McCord were appointed to read Essays; and Dr. York to deliver a public address, at the next meeting to be held in Paris, on the 3d Wednesday in October next.

CHARLES JOHNSTON, M. D., President.

S. W. THOMPSON, M. D, Secretary.

